# Inter­molecular inter­actions and disorder in six isostructural cele­coxib solvates

**DOI:** 10.1107/S2053229620008359

**Published:** 2020-06-27

**Authors:** Andrew D. Bond, Changquan C. Sun

**Affiliations:** a Department of Chemistry, University of Cambridge, Lensfield Road, Cambridge, CB2 1EW, England; b Department of Pharmacy, University of Copenhagen, Universitetsparken 2, Copenhagen, DK-2100, Denmark; c Department of Pharmaceutics, University of Minnesota, Minneapolis, MN 5545, USA

**Keywords:** cele­coxib, active pharmaceutical ingredient, API, solvate, crystal structure, isostructurality, disorder, *PIXEL*, anti-inflammatory

## Abstract

Six isostructural crystalline solvates of the active pharmaceutical ingredient cele­coxib {4-[5-(4-methyl­phen­yl)-3-(tri­fluoro­meth­yl)pyrazol-1-yl]benzene­sul­fon­amide; C_17_H_14_F_3_N_3_O_2_S} are described, containing di­methyl­formamide (DMF, C_3_H_7_NO, **1**), di­methyl­acetamide (DMA, C_4_H_9_NO, **2**), *N*-methylpyrrolidin-2-one (NMP, C_5_H_9_NO, **3**), tetra­methyl­urea (TMU, C_5_H_12_N_2_O, **4**), 1,3-dimethyl-3,4,5,6-tetra­hydro­pyrimidin-2(1*H*)-one (DMPU, C_6_H_12_N_2_O, **5**) or dimethyl sulfoxide (DMSO, C_2_H_6_OS, **6**). The host cele­coxib structure contains one-dimensional channel voids accommodating the solvent mol­ecules, which accept hydrogen bonds from the NH_2_ groups of two cele­coxib mol­ecules. The solvent binding sites have local twofold rotation symmetry, which is consistent with the point symmetry of the solvent mol­ecule in **4** and **5**, but introduces orientational disorder for the solvent mol­ecules in **1**, **2**, **3** and **6**. Despite the isostructurality of **1**–**6**, the unit-cell volume and solvent-accessible void space show significant variation. In particular, **4** and **5** show an enlarged and skewed unit cell, which can be attributed to a specific inter­action between an N—CH_3_ group in the solvent mol­ecule and the toluene group of cele­coxib. Inter­molecular inter­action energies calculated using the *PIXEL* method show that the total inter­action energy between the cele­coxib and solvent mol­ecules is broadly correlated with the mol­ecular volume of the solvent, except in **6**, where the increased polarity of the S=O bond leads to greater overall stabilization com­pared to the similarly-sized DMF mol­ecule in **1**. In the structures showing disorder, the most stable orientations of the solvent mol­ecules make C—H⋯O contacts to the S=O groups of cele­coxib.

## Abstract 2

Six isostructural crystalline solvates of cele­coxib are reported and the inter­molecular inter­actions involving the solvent mol­ecules are described.

## Introduction   

Understanding the structures and properties of crystalline solids can be of significant importance for active pharmaceutical ingredients (APIs) (Sun, 2009 ▸). Solid-form screening is an integral part of most pre-formulation activities (Morissette *et al.*, 2004 ▸), with an aim to establish the range of solid forms that can exist for a given API. These generally include both polymorphs and multicom­ponent forms, which may variously be described as salts, cocrystals, solvates, *etc*. (Aitipamula *et al.*, 2012 ▸).

The API of inter­est in this work is the anti-inflammatory drug cele­coxib (see Scheme 1). To date, there is only one polymorph (Form III) of cele­coxib for which a single-crystal X-ray structure has been reported (Dev *et al.*, 1999 ▸; Wang *et al.*, 2019 ▸) in the Cambridge Structural Database (CSD; Groom *et al.*, 2016 ▸), although several polymorphs are established in the literature (Dev *et al.*, 1999 ▸; Lu *et al.*, 2006 ▸; Wang & Sun, 2019 ▸). Crystal structures are known for numerous multicom­ponent forms, including with pyrrolidin-2-one, caprolactam, valerolactam (Bolla *et al.*, 2014 ▸), pyridin-2(1*H*)-one (Bolla & Nangia, 2019 ▸), nicotinamide (Zhang *et al.*, 2017 ▸) and bis­(proline) (as a zwitterion; Li *et al.*, 2018 ▸). Some cele­coxib cocrystals with 4,4′-bi­pyridine and 1,2-bis­(pyridin-4-yl)ethyl­ene have also been reported to form isostructural solvates (*i.e*. isostructural three-com­ponent crystals) when combined with acetone, THF or 1,4-dioxane (Wang *et al.*, 2014 ▸).

We were initially inter­ested in studying solvates of cele­coxib as part of a broader structure–property correlation exercise to understand and address its pharmaceutical deficiencies, *e.g.* high punch sticking propensity (Wang *et al.*, 2020 ▸; Paul *et al.*, 2017 ▸, 2020 ▸), amorphous phase stability (Wang & Sun, 2019 ▸), poor flowability (Chen *et al.*, 2020 ▸) and high elastic flexibility (Wang *et al.*, 2019 ▸). In the course of this work, we identified a group of six solvates (see Scheme 1) that form an isostructural group, different from any of the multicom­ponent cele­coxib crystal structures in the CSD. We describe the new structure type in this paper and explore aspects of the isostructurality, including variation of the unit-cell parameters, disorder of the solvent mol­ecules, and the inter­molecular inter­actions between the solvent and cele­coxib mol­ecules.

## Experimental   

### Synthesis and crystallization   

Single crystals suitable for X-ray analysis were obtained by slow cooling of a warm solution of cele­coxib in either di­methyl­formamide (DMF, **1**), di­methyl­acetamide (DMA, **2**), *N*-methylpyrrolidin-2-one (NMP, **3**), tetra­methyl­urea (TMU, **4**), 1,3-dimethyl-3,4,5,6-tetra­hydropyrimidin-2(1*H*)-one (DMPU, **5**) or dimethyl sulfoxide (DMSO, **6**). The DMF (**1**) and DMA (**2**) solvates have been prepared previously (Chawla *et al.*, 2003 ▸), but structural details were not provided. A powder X-ray diffraction pattern published by Chawla *et al.* (2003 ▸) clearly matches that simulated from the crystal structure of **2**. The match to **1** is less clear, but additional thermal analysis is broadly consistent with our observations, so it seems probable that the structures described herein are consistent with the previously studied material.
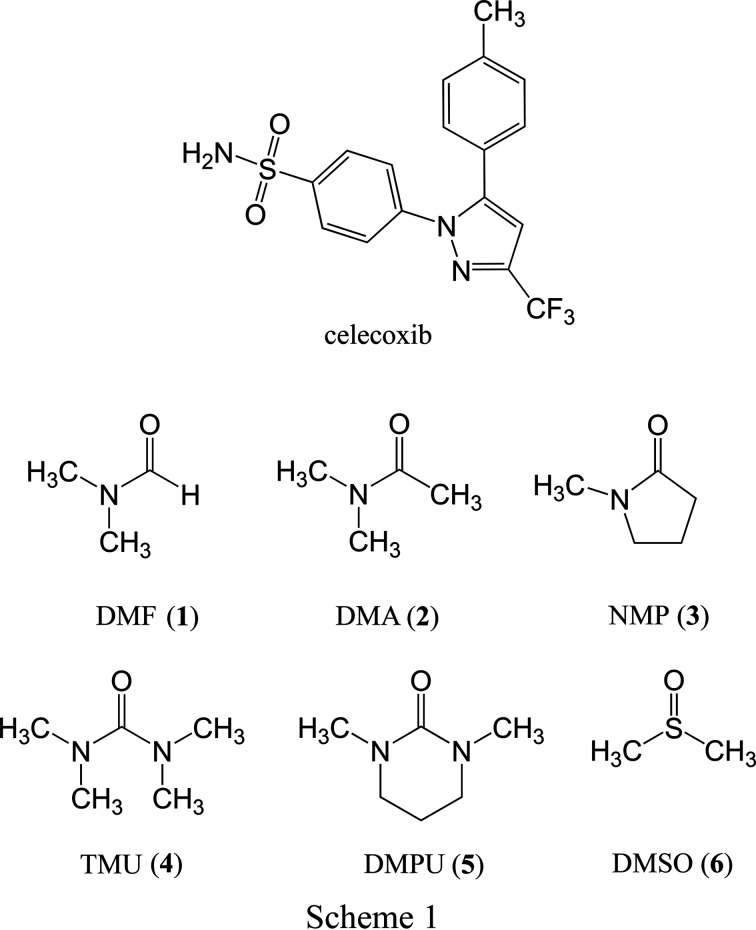


### Refinement of 1–6   

H atoms bound to C atoms were placed in idealized positions and refined using a riding model, with *U*_iso_(H) = 1.2 or 1.5*U*_eq_(C). For the methyl group (C11) in cele­coxib, the H atoms were allowed to rotate around the local threefold axis. H atoms of the NH_2_ groups were located in difference Fourier maps, then refined with isotropic displacement parameters, with the N—H and H⋯H distances restrained to 0.86 (1) and 1.50 (1) Å, respectively. All of the structures display rotational disorder of the CF_3_ group. This was modelled in each case as two sets of three F atoms, with site-occupancy factors constrained to sum to unity. To ensure a regular geometry, the C—F distances were restrained to a common refined value and the F⋯F distances were restrained to 1.633 times that value. All F atoms were refined with anisotropic ADPs. This produces highly distorted (prolate) ellipsoids in several cases, despite the inclusion of two sets of atomic sites, indicating that the rotational disorder is extensive. Given this rotational disorder, the distorted ellipsoids were considered to be an acceptable com­promise to model the electron density in this region. For the disordered solvent mol­ecules in **1**, **2**, **3** and **6**, two sets of atoms were refined, with site-occupancy factors summing to unity, and with appropriate geometrical restraints. Anisotropic ADPs were applied to all non-H atoms and H atoms were placed in idealized positions and refined as riding. The structure and refinement details are presented in Table 1 ▸.

### Computational details   

The crystal structures were energy-minimized with dispersion-corrected density functional theory (DFT-D) using the *CASTEP* module (Clark *et al.*, 2005 ▸) in *Materials Studio* (Accelrys, 2011 ▸). The PBE functional (Perdew *et al.*, 1996 ▸) was applied with a plane-wave cut-off energy of 520 eV, in combination with the Grimme semi-empirical dispersion correction (Grimme, 2006 ▸). The unit-cell parameters were constrained to the experimental values and the space group *P*2_1_/*c* was imposed. Only one com­ponent of the rotationally-disordered CF_3_ group was included (arbitrarily). For the structures with disordered solvent mol­ecules, separate models were minimized for each disorder com­ponent. In each case, minimization produced only small geometrical deviations from the starting structure, as expected for high-quality single-crystal structures (van de Streek & Neumann, 2010 ▸). The purpose of the optimization step is to place the structures on a common basis for com­parison, particularly for the disordered structures, where the results of the crystallographic refinement are generally less precise. The DFT-D-optimized structures were then used as input for the *PIXEL* module of the *CSP* package (Gavezzotti, 2002 ▸, 2003 ▸, 2011 ▸). The calculated pairwise inter­action energies are estimated to have an accuracy within a range of *ca* ±3 kJ mol^−1^.

## Results and discussion   

### Description of the crystal structures   

**Figure 1:**
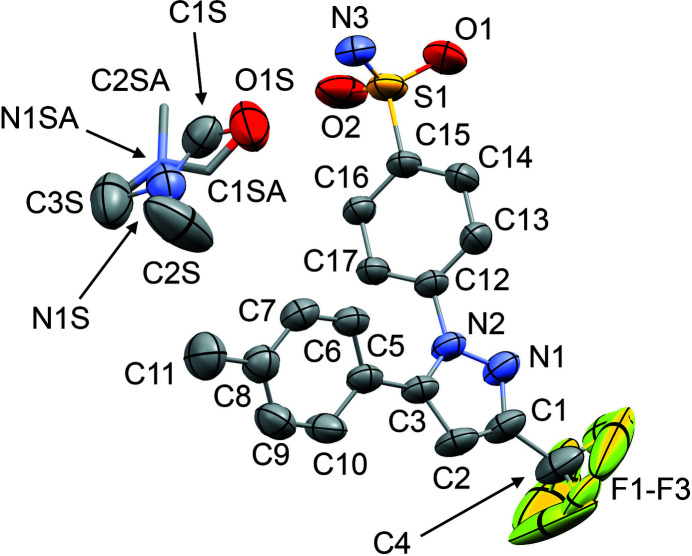
The mol­ecular structure of **1**, with displacement ellipsoids at the 50% probability level. H atoms have been omitted. The second disorder com­ponent of the DMF mol­ecule [site-occupancy factor = 0.221 (7)] is shown in outline only. Atoms O1*S* and C3*S* are common to both DMF com­ponents.

The mol­ecular structures of **1**–**6** are shown in Figs. 1 ▸–6 ▸ ▸ ▸ ▸ ▸. In the crystal structures, the cele­coxib mol­ecules are arranged into pairs around inversion centres with two solvent mol­ecules accepting N—H⋯O hydrogen bonds (Fig. 7 ▸). The local symmetry of this unit is effectively 2/*m* (*C*_2h_), where the C=O (or S=O) group of each solvent mol­ecule is approximately aligned along the local twofold rotation axis. For the TMU and DMPU mol­ecules in **4** and **5**, which themselves show twofold rotational point symmetry, this produces an ordered crystallographic result. The DMPU mol­ecule displays minor conformational disorder of its six-membered ring (atoms C3*S*/C3*SA* in Fig. 5 ▸), but the parts of the mol­ecule involved in binding to cele­coxib are ordered and consistent for TMU and DMPU. For DMF (**1**), DMA (**2**), NMP (**3**) and DMSO (**6**), which do not possess twofold rotational symmetry, the crystal structure is disordered, with the mol­ecules adopting two alternative orientations related by the local twofold axis. However, the full crystallographic environment of each solvent mol­ecule is not twofold symmetric. Hence, the two orientations of the disordered solvent mol­ecules have different total inter­action energies (§3.3). With the solvent mol­ecules removed from the structures, the void space between the cele­coxib mol­ecules defines one-dimensional (1D) channels along the *b* axis (Fig. 8 ▸). The solvent-accessible volume spans a considerable range for **1**–**6** (Table 2 ▸), constituting approximately 21–28% of the unit-cell volume.

### Variation of the unit-cell parameters   

Despite the isostructural nature of the solvates, the unit-cell parameters differ quite significantly, with a difference of *ca* 250 Å^3^ between the smallest (**6**) and largest (**4**) volumes. Plotting the *b* or *c* axis of **1**–**6** by ascending length (see supporting information) shows an approximately linear change in each case, but plotting the *a* axis in a similar manner shows a clear discontinuity, with the *a* axis in **4** and **5** being approximately 0.5 Å longer than in **1**, **2**, **3** and **6**. A similar pattern is seen for the β angle, indicating a relative skewing of the *ac* plane in **4** and **5**. Comparing representative structures in projection along the *b* axis (Fig. 9 ▸) indicates a reason for this observation. Common to structures **4** and **5**, but not present in **1**, **2**, **3** or **6**, is an N—CH_3_ group in the solvent mol­ecule that points approximately along the *a* axis and is directed towards a neighbouring toluene ring of cele­coxib. The inter­action pushes the toluene ring away from the position seen in the structures that do not have this N—CH_3_ group. The cele­coxib mol­ecules are ‘anchored’ by their hydrogen-bonding NH_2_ groups, which retain essentially identical positions in all structures, so the effect of pushing away the toluene ring is a relative rotation of the cele­coxib mol­ecules (Fig. 9 ▸). This serves to elongate both the *a* and the *c* axes, and to skew the unit cell. The other CH_3_ groups in TMU (**4**) or DMPU (**5**) adopt positions that are seen in one or more of the other structures, and they do not make any clearly com­parable inter­molecular contacts to cele­coxib.

### Inter­actions between the solvent mol­ecules and cele­coxib   

On account of the isostructurality, the pairwise inter­molecular inter­actions in each structure can be directly matched. Table 2 ▸ lists the total inter­action energy between the cele­coxib and solvent mol­ecules, based on an equivalent set of inter­actions in each structure. For **1**–**5**, the total cele­coxib–solvent inter­action energy broadly increases with the mol­ecular volume of the solvent, with **2** (DMA) and **3** (NMP) being closely com­parable. The DMSO mol­ecule in **6** has a significantly more stabilizing total inter­action with cele­coxib, com­pared to the similarly-sized DMF mol­ecule in **1**, due to the increased polarity of the S=O bond. For example, the two independent pairwise inter­actions including the hydrogen bonds to cele­coxib are both approximately −50 kJ mol^−1^ in **1** (varying slightly for the two disorder com­ponents), but approximately −57 and −68 kJ mol^−1^ in **6**. The N—CH_3_⋯π inter­action highlighted in Fig. 9 ▸ belongs to the cele­coxib–solvent pair within the asymmetric unit (as shown in Figs. 4 ▸ and 5 ▸). Since *PIXEL* energies refer to total pairwise inter­molecular inter­actions, any specific features of the N—CH_3_⋯π inter­action are masked by the total inter­action energy. Table 2 ▸ also lists the total inter­action energy between solvent mol­ecules, based on three equivalent significant inter­actions in each structure. The inter­action between the two solvent mol­ecules involved in the hydrogen-bonded motif (Fig. 7 ▸) is repulsive, due to the destabilizing Coulombic O⋯O inter­action. The only other significant inter­actions between solvent mol­ecules are along the 2_1_ screw axis parallel to *b*, which are slightly stabilizing. The extent to which these inter­actions mitigate the destabilizing O⋯O inter­action increases with the mol­ecular volume of the solvent, and the overall solvent–solvent inter­action is slightly stabilizing for the largest mol­ecule, *i.e.* DMPU (**5**).

**Figure 2:**
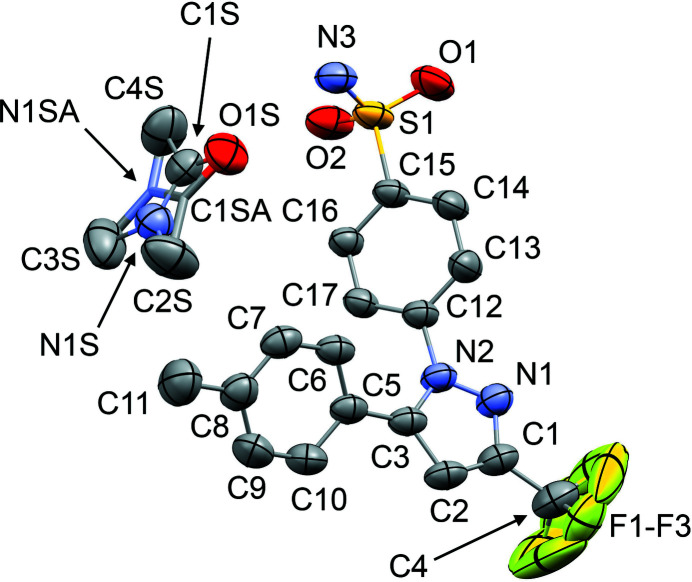
The mol­ecular structure of **2**, with displacement ellipsoids at the 50% probability level. H atoms have been omitted. The second disorder com­ponent of the DMA mol­ecule [site-occupancy factor = 0.464 (8)] is shown in outline only. Atoms O1*S*, C2*S*, C3*S* and C4*S* are common to both DMA com­ponents.

**Figure 3:**
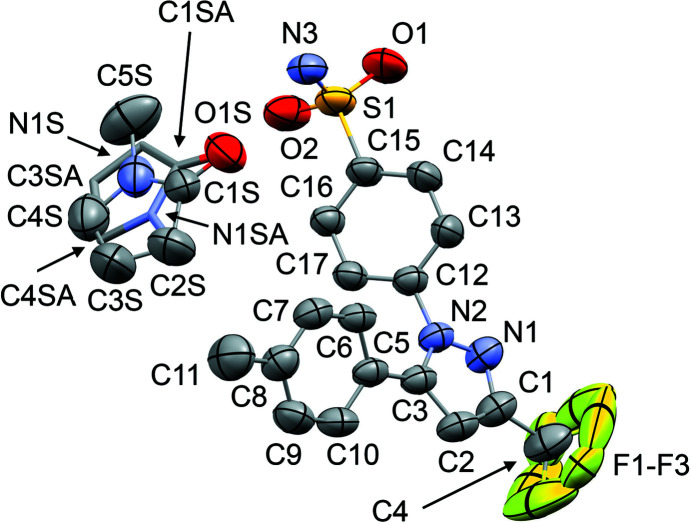
The mol­ecular structure of **3**, with displacement ellipsoids at the 50% probability level. H atoms have been omitted. The second disorder com­ponent of the NMP mol­ecule [site-occupancy factor = 0.321 (8)] is shown in outline only. Atoms C2*SA* and C5*SA* are not labelled, C2*SA* is directly below C5*S* and C5*SA* is hidden behind C2*S*.

**Figure 4:**
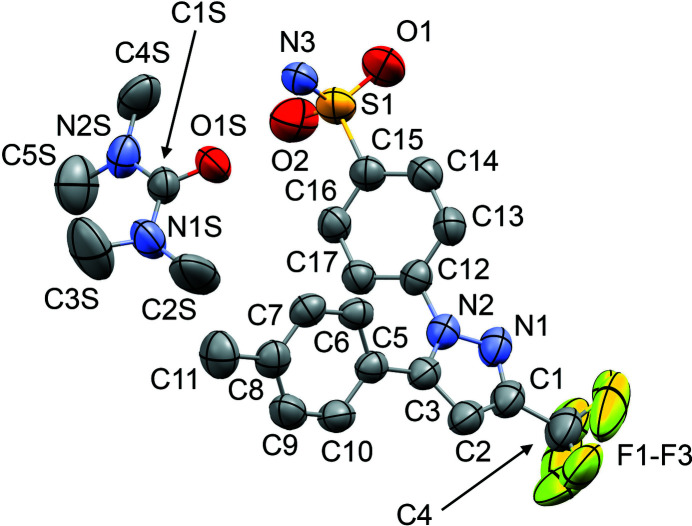
The mol­ecular structure of **4**, with displacement ellipsoids at the 50% probability level. H atoms have been omitted. Disorder is not evident for the TMU mol­ecule.

**Figure 5:**
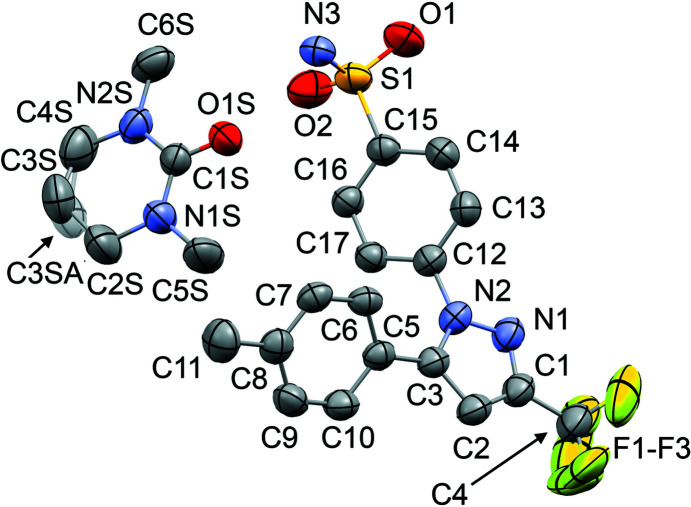
The mol­ecular structure of **5**, with displacement ellipsoids at the 50% probability level. H atoms have been omitted. The alternative positions C3*S* and C3*SA* [site-occupancy factors = 0.584 (16):0.416 (16)] are shown for the DMPU mol­ecule.

**Figure 7:**
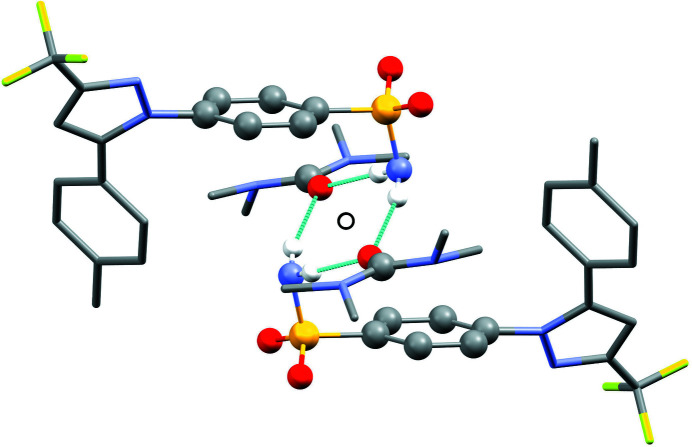
Hydrogen-bonded motif with two solvent mol­ecules (TMU is shown) accepting N—H⋯O hydrogen bonds from two cele­coxib mol­ecules across a crystallographic inversion centre (indicated by the open circle). H atoms not involved in hydrogen bonding have been omitted. The atoms shown in ball-and-stick style conform to local 2/*m* (*C*_2h_) point symmetry.

**Figure 8:**
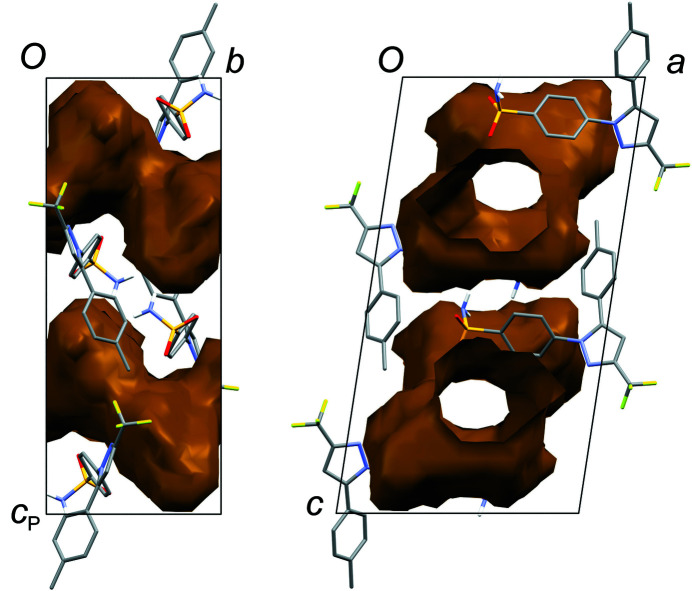
Views of the 1D voids running along the *b* axis in the cele­coxib framework structure, after removing the solvent mol­ecules. Structure **6** is shown. The voids are generated using *Mercury* (contact surface, probe radius 1.2 Å; Macrae *et al.*, 2020 ▸).

**Figure 9:**
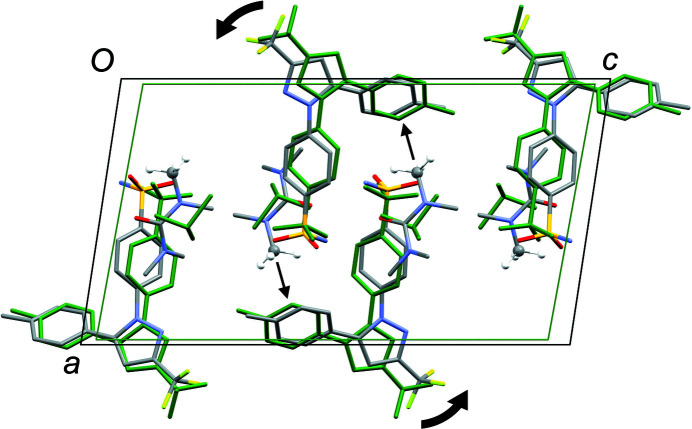
Projection of the structures of **4** (standard colour) and **6** (green) along the *b* axis. The highlighted N—CH_3_⋯π inter­action in **4** [C2*S*⋯centroid(C5–C10) = 3.825 Å] causes the cele­coxib mol­ecules to rotate outwards relative to each other, as indicated by the thick arrows, causing expansion and skewing of the unit cell com­pared to **6**.

**Figure 10:**
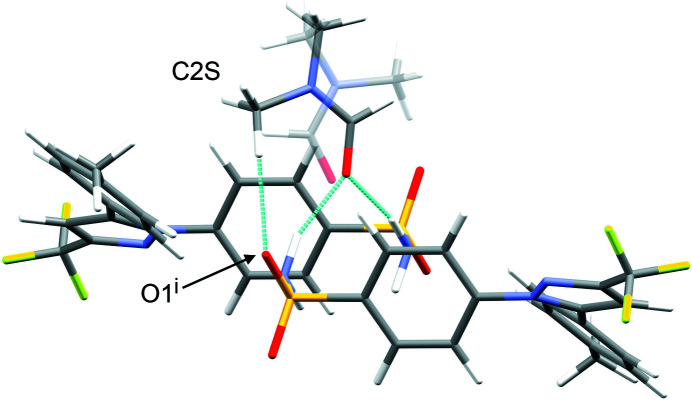
The most stable orientation of the disordered DMF mol­ecule in **1**, high­lighting the C—H⋯O contact to an S=O group of cele­coxib [symmetry code: (i) −*x* + 1, −*y* + 1, −*z* + 1]. The less stable DMF orientation is shown as semi-transparent.

**Figure 11:**
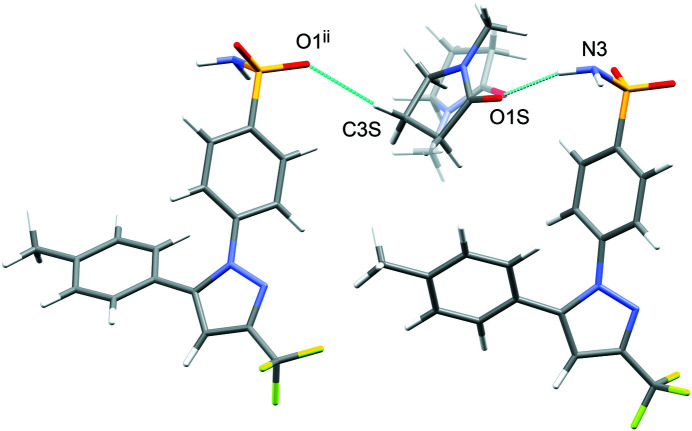
The most stable orientation of the disordered NMP mol­ecule in **3**. A short C—H⋯O contact is made to an S=O group of the neighbouring cele­coxib mol­ecule [symmetry code: (ii) *x*, −*y* + 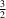, *z* − 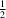]. The less stable NMP orientation for **3** is shown as semi-transparent. A com­parable C—H⋯O contact is seen for the most stable disorder com­ponent of DMPU in **5**.

The difference between the total inter­action energies with the cele­coxib framework for the two disorder com­ponents of the solvent mol­ecules in each structure is also shown in Table 2 ▸. The most significant difference within a single structure is seen for DMF (**1**), where the two orientations exchange the positions of the C—H and N—CH_3_ groups (Fig. 1 ▸). Approximately two thirds of the energy difference arises from the inter­actions of the DMF mol­ecule with the two cele­coxib mol­ecules in the hydrogen-bonding motif (Fig. 7 ▸), where the more stable DMF orientation brings the CH_3_ group of atom C2*S* into close proximity to O1 in one of the S=O bonds (Fig. 10 ▸). For DMA (**2**), the disorder com­ponents have essentially the same mol­ecular footprint (the positions of atoms O1*S*, C2*S*, C3*S* and C4*S* are common to both DMA com­ponents; Fig. 2 ▸), and the total inter­action energies of the two com­ponents with the cele­coxib framework are the same within the expected precision of the calculations. For NMP (**3**), the two solvent orientations are geometrically com­parable, except for the positions of C3*S*/C3*SA* (Fig. 3 ▸). However, one orientation is noticeably more stable than the other. A significant energy difference also exists for the two orientations of the DMPU mol­ecule in **5**, for which the principal geometrical difference is the position of one CH_2_ group (C3*S*/C3*SA*), with accom­panying differences in the positions of the H atoms on the neighbouring CH_2_ groups. Comparing the most stable disorder com­ponents for **3** and **5**, they share a common position for one CH_2_ group (C3*S* in **3** and C2*S* in **5**) that is not seen in the other disorder com­ponents. This introduces a short C—H⋯O contact to an S=O group of the neighbouring cele­coxib mol­ecule (Fig. 11 ▸). The *PIXEL* energies confirm that the inter­action with this cele­coxib mol­ecule is significantly more stabilizing when this contact is present than when it is not. For the DMSO mol­ecules in **6**, the difference between the two solvent orientations involves only the position of the S atom, and the inter­action energies with the cele­coxib framework are com­parable.

## Conclusion   

This set of six isostructural cele­coxib solvates includes small solvent mol­ecules that can accept hydrogen bonds. The host cele­coxib framework is consistent within the set, but it shows quite substantial flexibility in its unit-cell parameters and solvent-accessible void space, and can therefore accommodate solvent mol­ecules of varying size and shape. The crystallographic disorder in several of the structures is understandable on the basis of the local twofold symmetry of the solvent binding site, com­pared to the point symmetry of the solvent mol­ecules. In the absence of any additional hydrogen-bond donors in the solvent mol­ecules, the next most stabilizing inter­actions between the solvent mol­ecules and the cele­coxib framework are C—H⋯O contacts to the S=O groups. The consideration of *PIXEL* inter­action energies, in combination with geometrical analysis of the crystal structures, is helpful in drawing these conclusions.

## Supplementary Material

Crystal structure: contains datablock(s) 1, 2, 3, 4, 5, 6, global. DOI: 10.1107/S2053229620008359/sk3751sup1.cif

Structure factors: contains datablock(s) 1. DOI: 10.1107/S2053229620008359/sk37511sup2.hkl

Structure factors: contains datablock(s) 2. DOI: 10.1107/S2053229620008359/sk37512sup3.hkl

Structure factors: contains datablock(s) 3. DOI: 10.1107/S2053229620008359/sk37513sup4.hkl

Structure factors: contains datablock(s) 4. DOI: 10.1107/S2053229620008359/sk37514sup5.hkl

Structure factors: contains datablock(s) 5. DOI: 10.1107/S2053229620008359/sk37515sup6.hkl

Structure factors: contains datablock(s) 6. DOI: 10.1107/S2053229620008359/sk37516sup7.hkl

Unit-cell volume and a/b/c/beta parameter plots. DOI: 10.1107/S2053229620008359/sk3751sup8.pdf

Supporting information file. DOI: 10.1107/S2053229620008359/sk37511sup9.cml

Supporting information file. DOI: 10.1107/S2053229620008359/sk37512sup10.cml

Supporting information file. DOI: 10.1107/S2053229620008359/sk37513sup11.cml

Supporting information file. DOI: 10.1107/S2053229620008359/sk37514sup12.cml

Supporting information file. DOI: 10.1107/S2053229620008359/sk37515sup13.cml

Supporting information file. DOI: 10.1107/S2053229620008359/sk37516sup14.cml

Additional CIFs (DFT calculations). DOI: 10.1107/S2053229620008359/sk3751sup15.txt

CCDC references: 2011633, 2011634, 2011635, 2011636, 2011637, 2011638

## Figures and Tables

**Figure 6:**
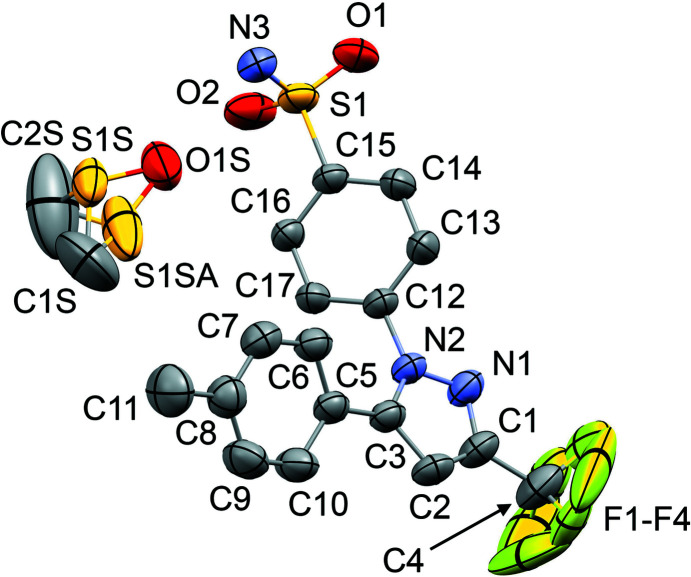
The mol­ecular structure of **6**, with displacement ellipsoids at the 50% probability level. H atoms have been omitted. Atoms C1*S* and C2*S* are common to both disorder com­ponents for the DMSO mol­ecule. The relatively large displacement ellipsoid of atom C2*S* was retained in preference to multiple atom sites for simplicity of the model.

**Table:** For all structures: monoclinic, *P*2_1_/*c*, *Z* = 4. Experiments were carried out at 298 K with Cu *K*α radiation using a Bruker D8-QUEST PHOTON-100 diffractometer. Absorption was corrected for by multi-scan methods (*SADABS*; Bruker, 2016 ▸). H atoms were treated by a mixture of independent and constrained refinement.

	**1**	**2**	**3**
Crystal data			
Chemical formula	C_17_H_14_F_3_N_3_O_2_S·C_3_H_7_NO	C_17_H_14_F_3_N_3_O_2_S·C_4_H_9_NO	C_17_H_14_F_3_N_3_O_2_S·C_5_H_9_NO
*M*_r_	454.47	468.49	480.50
*a*, *b*, *c* (Å)	11.8973 (4), 8.8360 (3), 21.8286 (7)	11.9584 (3), 9.2028 (2), 21.2811 (6)	11.9978 (4), 9.0896 (3), 21.9732 (8)
β (°)	103.4537 (13)	103.3826 (12)	101.358 (2)
*V* (Å^3^)	2231.75 (13)	2278.41 (10)	2349.36 (14)
μ (mm^−1^)	1.77	1.75	1.71
Crystal size (mm)	0.16 × 0.16 × 0.14	0.20 × 0.18 × 0.18	0.20 × 0.18 × 0.18

Data collection			
*T*_min_, *T*_max_	0.440, 0.753	0.608, 0.753	0.593, 0.753
No. of measured, independent and observed [*I* > 2σ(*I*)] reflections	16627, 3940, 3362	38939, 4032, 3394	24378, 4146, 3112
*R*_int_	0.053	0.041	0.047
(sin θ/λ)_max_ (Å^−1^)	0.596	0.597	0.596

Refinement			
*R*[*F* ^2^ > 2σ(*F* ^2^)], *wR*(*F* ^2^), *S*	0.056, 0.149, 1.08	0.047, 0.129, 1.03	0.048, 0.136, 1.03
No. of reflections	3940	4032	4146
No. of parameters	346	345	389
No. of restraints	35	26	103
Δρ_max_, Δρ_min_ (e Å^−3^)	0.25, −0.37	0.22, −0.29	0.22, −0.24

**Table:** 

	**4**	**5**	**6**
Crystal data			
Chemical formula	C_17_H_14_F_3_N_3_O_2_S·C_5_H_12_N_2_O	C_17_H_14_F_3_N_3_O_2_S·C_6_H_12_N_2_O	C_17_H_14_F_3_N_3_O_2_S·C_2_H_6_OS
*M*_r_	497.54	509.55	459.50
*a*, *b*, *c* (Å)	12.4050 (3), 8.9351 (2), 22.5727 (6)	12.4495 (17), 8.7822 (13), 22.656 (3)	11.9884 (3), 9.0230 (3), 20.8537 (6)
β (°)	98.6702 (13)	97.861 (5)	100.3908 (9)
*V* (Å^3^)	2473.36 (11)	2453.9 (6)	2218.78 (11)
μ (mm^−1^)	1.66	1.68	2.63
Crystal size (mm)	0.16 × 0.14 × 0.14	0.20 × 0.20 × 0.18	0.14 × 0.12 × 0.12

Data collection			
*T*_min_, *T*_max_	0.657, 0.753	0.526, 0.753	0.476, 0.753
No. of measured, independent and observed [*I* > 2σ(*I*)] reflections	27591, 4397, 3339	25201, 4318, 3733	22464, 3916, 3520
*R*_int_	0.034	0.031	0.042
(sin θ/λ)_max_ (Å^−1^)	0.597	0.597	0.596

Refinement			
*R*[*F* ^2^ > 2σ(*F* ^2^)], *wR*(*F* ^2^), *S*	0.046, 0.142, 1.04	0.040, 0.118, 1.02	0.044, 0.126, 1.04
No. of reflections	4397	4318	3916
No. of parameters	349	364	318
No. of restraints	15	29	15
Δρ_max_, Δρ_min_ (e Å^−3^)	0.42, −0.20	0.25, −0.21	0.29, −0.28

Computer programs: *APEX3* (Bruker, 2018 ▸), *SAINT* (Bruker, 2018 ▸), *SHELXT* (Sheldrick, 2015*a* ▸), *SHELXL2018* (Sheldrick, 2015*b* ▸) and *Mercury* (Macrae *et al.*, 2020 ▸).

**Table 2:** Inter­molecular inter­action energies between the cele­coxib and solvent mol­ecules [*E*(tot)_cel–solv_] and between the solvent mol­ecules [*E*(tot)_solv–solv_], calculated using the *PIXEL* method, together with the unit-cell volume (*V*_cell_), void volume (*V*_void_) and solvent mol­ecular volume (*V*_solv_)

	Solvent	*V*_cell_ (Å^3^)	*V*_void_ (Å^3^)*^*a*^*	*V*_solv_ (Å^3^)*^*b*^*	Disorder com­ponent	*E*(tot)_cel–solv_ (kJ mol^−1^)	*E*(tot)_solv–solv_ (kJ mol^−1^)
**1**	DMF	2231.8	485.4 (21.7%)	76.7	**A**	−144.0	+3.8
					**B**	−132.2	+5.1
**2**	DMA	2278.4	481.8 (21.1%)	93.2	**A**	−160.5	+2.4
					**B**	−162.1	+3.3
**3**	NMP	2349.4	617.5 (25.0%)	100.1	**A**	−170.7	+1.9
					**B**	−160.1	+1.5
**4**	TMU	2473.4	572.3 (24.4%)	121.8	–	−155.1	+0.7
**5**	DMPU	2453.9	692.1 (28.2%)	127.9	**A**	−180.5	−1.9
					**B**	−187.1	−3.0
**6**	DMSO	2218.8	466.5 (21.0%)	71.7	**A**	−168.9	+4.6
					**B**	−164.3	+4.5

Notes: (*a*) the voids were generated using *Mercury* (Macrae *et al.*, 2020 ▸) as a contact surface with probe radius of 1.2 Å and (*b*) mol­ecular volumes are derived from van der Waals surfaces, calculated in *Materials Studio* (Accelrys, 2011 ▸) as a Connolly surface generated with zero probe radius.
