# Supplementary material for: Inter­molecular inter­actions and disorder in six isostructural cele­coxib solvates
Source: Acta Crystallogr C Struct Chem. 2020 Jun 27;76(Pt 7):632–8. doi: 10.1107/S2053229620008359 (PMC7336170; doi:10.1107/S2053229620008359)
Supplement: Supplementary file 8 [file c-76-00632-sup8.pdf]

STRUCTURAL
CHEMISTRY

Volume 76 (2020)

Supporting information for article:

Intermolecular interactions and disorder in six isostructural celecoxib solvates

Andrew D. Bond and Changquan C. Sun

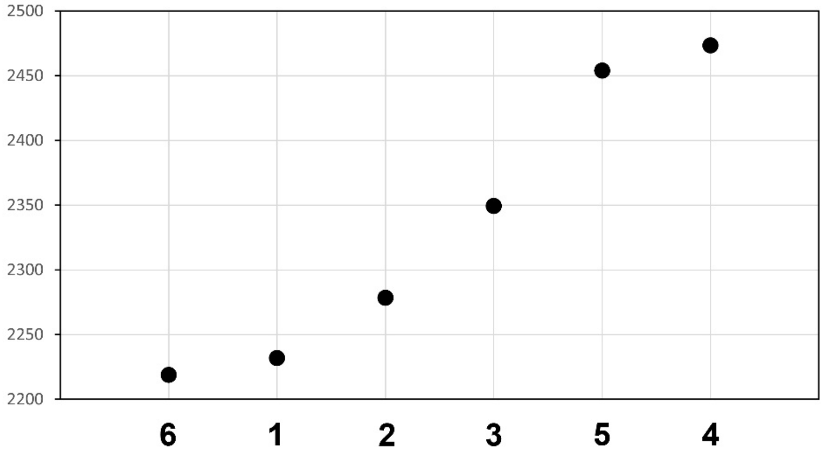

Figure S1 Plot of ascending unit-cell volume for structures **1–6**

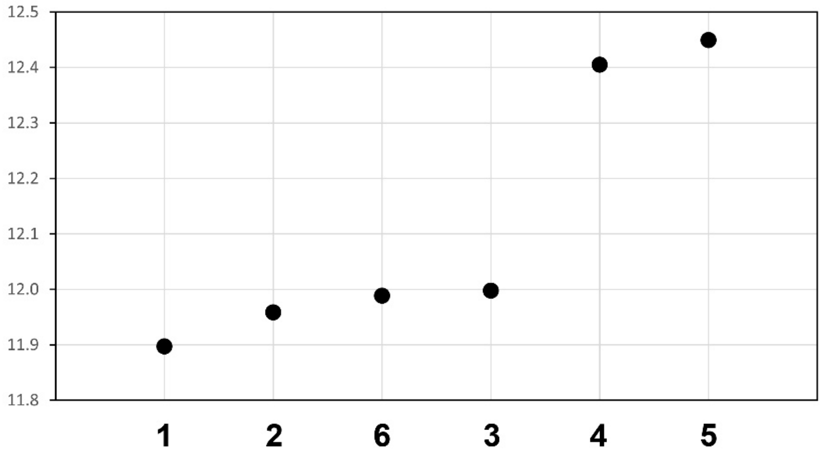

Figure S2 Plot of the *a* axis in ascending length order for structures **1**–**6**

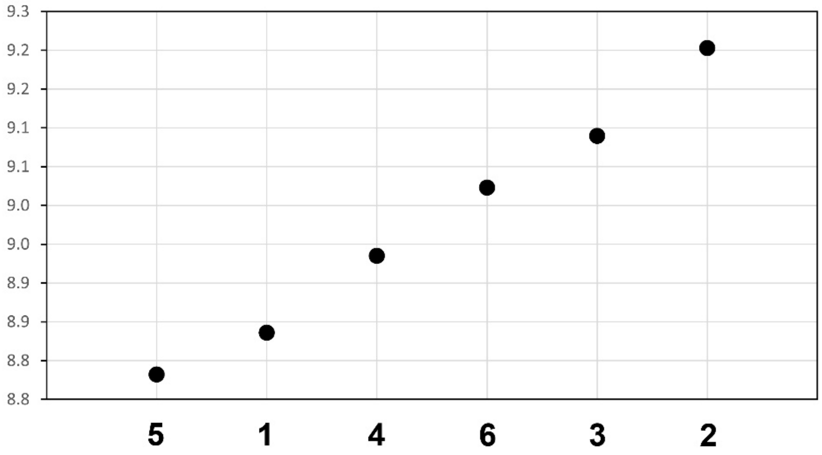

Figure S3 Plot of the *b* axis in ascending length order for structures **1**–**6**

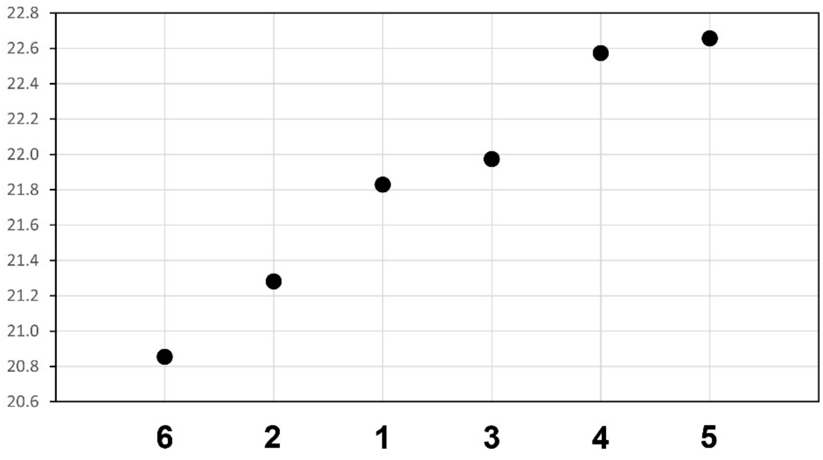

Figure S4 Plot of the *c* axis in ascending length order for structures **1**–**6**

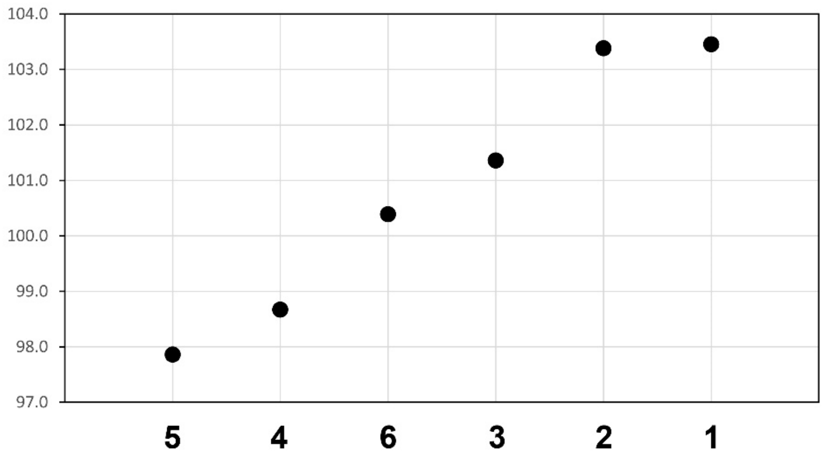

Figure S5 Plot of the β angle in ascending length order for structures **1**–**6**
